# Supplementary material for: Identification of photoperiod-induced specific miRNAs in the adrenal glands of Sunite sheep (Ovis aries)
Source: Front Vet Sci. 2022 Jul 22;9:888207. doi: 10.3389/fvets.2022.888207 (PMC9354845; doi:10.3389/fvets.2022.888207)
Supplement: Supplementary file 1 [file Data_Sheet_1.ZIP › Supplementary file/miRDeep2 file.zip!/miRDeep2 file/Know_Structure/SP42A1/oar-mir-377.pdf]

miRBase precursor : oar-mir-377
Total read count : 50
oar-miR-377-5p read count : 18
oar-miR-377-3p read count : 27
remaining reads : 5

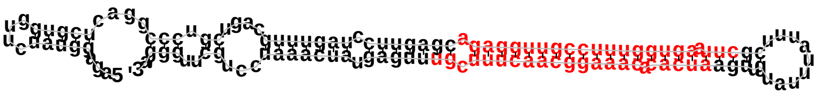

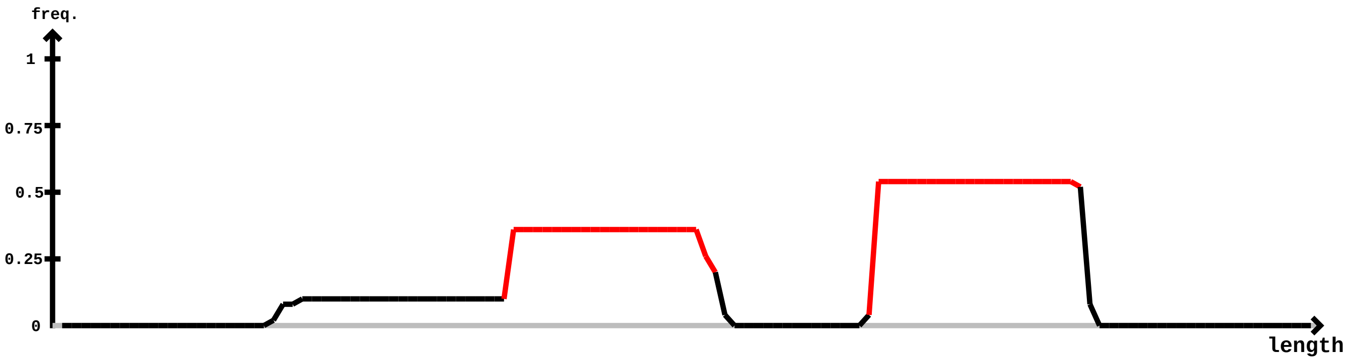

oar-miR-377-3p
oar-miR-377-5p

```
5'- agagguaucuuggugcucaggcccugcugacguuugauccuugagcagagguugccuuuggugaauucgcuuuauuuauguugaaucacacaaaggcaacuuucguuugaguaucaaauccugcuuggga   -3'    exp
    ...(((((....)))))....(((.((....(((((((.((((((.((((((((((((((((.(((((.........).))))))))).))))))))))))).))))))).)))))))...))..))).    reads      mm
    .....................cccugcugacguuugauccuugagc....................................................................................    1          0
    ......................ccugcugacguuugauccuugagc....................................................................................    3          0
    ........................ugcugacguuugauccuugagc....................................................................................    1          0
    ..............................................agagguugccuuuggugaau................................................................    5          0
    ..............................................agagguugccuuuggugaauu...............................................................    3          0
    ..............................................agagguugccuuuggugaauuc..............................................................    8          0
    ..............................................agagguugccuuuggugaauucg.............................................................    2          0
    ...................................................................................aaucacacaaaggcaacuuucgu........................    2          0
    ....................................................................................aucacacaaaggcaacuuucg.........................    1          0
    ....................................................................................aucacacaaaggcaacuuucgu........................    20         0
    ....................................................................................aucacacaaaggcaacuuucguu.......................    4          0
```
